# 

**Published:** 1879-05

**Authors:** 


### CONTENTS.

ORIGINAL.

ART. I.—The Anatomy and Dislocation of the Shoulder-joint. A paper read before the Rochester Pathological Society. By Wm. F. Sheehan, M. D., Rochester, N. Y....... 361
ART. II.—Causes of Diphtheria. By Henry Mills, Microscopist,...................... 366
ART. III.—Extirpation of the Spleen, reported by Henry W. Rogers,.................... 367

MISCELLANEOUS.

Amputation of the Femur,.................... 369
Prolapsus Ani,.................... 370
Uterine Polypi and Febroids,.................... 371
Amputation of the Cervix Uteri,.............. 373
Complimentary Dinner to Dr. S. D. Gross,.... 377
Some Practical Points in Operating for Cataract, 381
Treatment of Diphtheria,.................... 385
On the Biliary Secretion of the Dog with reference to the action of Cholagogues,........ 386
Free Dispensaries,.................... 388
The Adulteration of Drugs,.................... 389
Scarlet Fever in Newport, R. I.,.............. 389
The Board of Health Resolutions,............ 390
Syphilitic Consumption,.................... 390
Chlor-Acetic Acid in Cancer,.................. 390

EDITORIAL.

Employment of Female Assistant Physicians in female wards of Insane Asylums,....... 391
Buffalo College of Rational Medicine,.......... 393
Fare to the American Medical Association,... 393
Chicago Medical College,.................... 393
American Medical Association,................ 394
Laryngology. By Dr. Elsberg, .............. 394
Dialyzed Iron,.................... 394
Death of Dr. Waddel of Toledo,.............. 394
Books Reviewed,.................... 395
Books Received,.................... 398
Pamphlets,.................... 400

